# The nutritional state of children and adolescents with cerebral palsy is associated with oral motor dysfunction and social conditions: a cross sectional study

**DOI:** 10.1186/s12883-016-0573-8

**Published:** 2016-04-26

**Authors:** Vanessa Vieira Pinto, Levy Anderson César Alves, Fausto M. Mendes, Ana Lídia Ciamponi

**Affiliations:** Department of Orthodontics and Paediatric Dentistry, School of Dentistry, University of São Paulo (USP), Avenida Professor Lineu Prestes 2227, SP São Paulo, 05508-000 Brazil

**Keywords:** Children, Cerebral palsy, Nutrition, Oral motor function

## Abstract

**Background:**

Cerebral palsy (CP) is the main cause of severe physical impairment during childhood and has commonly shown oral motor association. It has been considered as the main cause of the high prevalence of problems in children’s nutrition. Respiration, chewing, swallowing, speaking and facial expressionare part of the orofacial motor functions and when affected they can interfere in children’s well-being. The aim of this study was to correlate two methods of orofacial motor evaluation, analyze the influence of orofacial motor functional impairment on the nutritional status of children and adolescents with CP, and the association between socioeconomic factors.

**Methods:**

Seventy children and adolescents with CP were selected, age range 6–16 years and following the exclusion criteria previously determined; 129 normoreactive children (control group), sex and age-matched to patients with CP. For the orofacial motor analysis two evaluation instruments were applied, the “Oral Motor Assessment Scale” (OMAS) and “Nordic Orofacial Test-Screening” (NOT-S). The anthropometric evaluation was based on the World Health Organization (WHO) and followed the criteria recommended by the Brazilian Ministry of Health.

**Results:**

There was statistically significant correlation between the oral motor methods of evaluation (*r* = -0.439, *p* < 0.0001). Concerning the nutritional status evaluation, being overweight was associated with dystonic and mixed CP forms variables (*p* = 0.034), mother with no partnership (*p* = 0.045) and mild oral motor impairment (*p* = 0.028).

**Conclusion:**

It could be concluded that, the weight’s gain by children and adolescents might be favored by a better functional oral motor performance and social factors.

## Background

Cerebral palsy (CP) is a term used to describe a variety of non-progressive disorders of posture and movement associated to immature brain defect [1]. Motor, growth and nutritional disorders are very common in CP patients, and some factors such as malnutrition and endocrine disturbs have been reported in the literature as common conditions influencing patient’s growth [2, 3].

Malnutrition has been considered a major worldwide problem and according to Dahlseng et al. [4] only 63 % of children with CP have normal Body Mass Index (BMI), with 16 % being overweight or obese and 20 % with thinness or severe thinness. Children with CP used to be considered small for their age, however, recently such condition seems to be changed in many countries.

Rogozinski et al. [5] reported an increase of 8.8 % in the prevalence of obesity among ambulatory children with CP from 1994–1997 to 2003–2004 in the US. Park et al. (2011) [6] also reported an increase in the prevalence of obesity among CP children in Korea, but lower than that observed from the US. Feeding problems are common associated with a poor linear growth. Few studies documented the association between chewing and swallowing problems and nutritional intake and growth [7]. Oral motor impairment may also potentially affect the functional capacity of children and their health quality of life [8]. Hence, studies relating the association between oral motor impairment and nutrition in children/adolescents with CP must be reported, so as to support professionals when dealing with pathology.

It has been hypothised that there is a correlation between oral motor dysfunction and gross motor dysfunction and that it correlates with under nutrition, but to our knowledge there are no published studies assessing the association between oral motor dysfunction and nutritional status.

Therefore, the aim of this study was to evaluate the association of functional motor orofacial performance with the orofacial nutritional status of children/adolescents with CP, evaluate the association between socioeconomic conditions, motor orofacial function and nutritional status, andthe correlation between two different motor assessment scales.

## Methods

This is a cross-sectional study that was conducted at the University of São Paulo–Dental School, from January to December 2013. The study was conducted after obtaining ethical clearance from the Ethics in Research Committee of the Dental School - University of São Paulo/BR (protocol n. 208/2010), according to the Helsinki Declaration.

A total of 110 children and adolescents, with age range 6–16 years with a clinical medical diagnosis of CP and attending the CAPE (Center of Attendance for Special Needs Patients) of the Dental School, University of São Paulo, Brazil, were potentially eligible to participate in the study. From that, those who were neurological immature and were not able to cooperate with the evaluation, with any chronic illness affecting growth other than CP, those who went through surgical procedures such as selective dorsal ryzotomy, intratecal baclofen or botulin toxine treatment as well as those parents of children/adolescents who did not accept to participate in the study were excluded. Therefore, participants included 70 children/adolescents with CP (study group) age- and sex-matched to 129 clinically healthy children/adolescents whose parents accepted to participate in the study (control group) and signed the written informed consent.

A questionnaire referring to demographic and socioeconomic data [9], as well as to general conditions of children/adolescents was administered to parents. Patients were clinically examined and their motor limitation was classified according to the Gross Motor Function Classification System (GMFCS) [10].

Some instruments of oral motor evaluation have been developed in search of knowledge of the oral motor function, to indicate affected area, evaluate intervention results, improve health attention and support the development of advanced treatment. Although the considerable effort aiming to establish ordinary criteria for this evaluation, the instruments available are still not used with frequency, for still being an area with very few studies.

In the present study, two instruments previously developed for the same aim were applied and correlated between themselves, as well as associated to the nutritional status of CP children and adolescents.

One of the instruments used was the NOT-S, though currently only a few studies have been available in the literature referring to the measurement of orofacial motor impairment [11–14] and none has been related to nutrition on CP individuals.

The OMAS was the other instrument used for this investigation, and was developed after the NOT-S, and maybe for that reason, there is still a little about this method. There is one study between young individuals with spastic cerebral palsy that investigated the influence of orofacial motor performance, given by the OMAS, nutritional status and salivary parameters. The complexity of those oral functional instruments is different and both have advantages and disadvantages.

With regards to the NOT-S instrument, it showed to be better than the OMAS as it does not involve meal costs, an advantage for population studies and/or for those studies which are developed in more distant areas and with little resources. In that case, people would need specific places to obtain food, and also adequate facilities to store them, for instance the yoghurt used for evaluation.

Though, the OMAS offered the advantage that it did not depend on the cooperation and direct interaction of the patient, once the examiner observes the individual during the repetition of daily actions when eating. The time spent during the evaluation process was not recorded, but it can be inferred that when using the OMAS the examiner spends less time and it occurs due to the smaller number of items evaluated and also because individuals do not have to understand how movements must be done.

Both instruments were chosen for the study due to their efficiency on identifying which areas of the oral motor function have been harmed. Besides, it is also possible to evaluate different levels of impairment comparing both groups (study and control), also, both of them meet high reliability standards and are translated and validated into Portuguese [13, 15].

We evaluated whether there was any correlation between the instruments, since both aim to evaluate the same oral motor functions. This is the first study that correlates OMAS and NOT-S.

Patient oral motor performance was evaluated during the feeding process using the Brazilian validated version of NOT-S [16–18] and OMAS [15] (Kappa intraexamine *r* = 0.94 and Kappa intraexamine *r* = 0.96, respectively).

The NOT-S consists of a structured interview and a clinical examination, each comprising six domains: Interview - sensory function, breathing, habits, chewing and swallowing, drooling and the dryness of the mouth. Clinical examination – face at rest, nose breathing, facial expression, masticatory muscle and jaw function, oral motor function and speech [16].

For the OMAS, the following issues were evaluated: mouth closure, lip closure onto the utensils, lip closure during deglutition, control of food during swallowing (solid/semisolid), mastication, straw suction and control of liquids during deglutition. The final result was given by a score for each topic of the oral motor skill assessed [15].

The anthropometric evaluation was based on the WHO growth standards [16], following the criteria recommended by the Brazilian Ministry of Health, Feeding and Nutritional Surveillance System (SISVAN) [19] and recorded on the clinical form.

In order to evaluate the body weight of children/adolescents with CP, the caregiver was weighed and the body mass was recorded in the evaluation form. Afterwards, the caregiver holding the child/adolescent was weighed and the total weight was recorded. The final weight was calculated by subtracting the total value and the caregiver weight. The height of CP patients was estimated, since they can present osteo-skeletal malformations, muscle spasms, cognitive impairment, disorder of equilibrium and seizures. Measurements of long bones were performed according to Stevenson’s [20] methodology (Knee height × 2.69/24.2). The knee height was measured using the Hardenden Antropometer with the knee and ankle being bent at a 90^0^). For typically developing children, the height was measured by means of a height rule (Welmy®, W200A, Brazil).

The evaluation of the nutritional status was made by means of the Body Mass Index (BMI) Z-score and calculated by the AnthroPlus [21] software. The WHO and SISVAN standards propose the following cut-offs: overweight: > + 1SD, obesity: > + 2SD, severe obesity > +3 SD, thinness: <- 2SD, severe thinness: <-3SD.

### Statistical analysis

In order to correlate the evaluation methods of oral motor function, the Spearman correlation coefficient was calculated. The association between the methods and some independent variables (group, types of CP, and GMFCS) was evaluated by using the Poisson regression.

Concerning the outcomes of oral motor function and nutritional status, the findings were: overweight (eutrophy + thinness + severe thinness versus overweight + obesity + severe obesity) and underweight (eutrophy + overweight + obesity + severe obesity versus thinness + severe thinness). The independent variables (socioeconomic and clinical features of children/adolescents) tested were: OMAS, NOT-S, groups, form of CP (spastic versus dystonic + mixed), GMFCS, use of medication, mother’s marital status (with or without partnership).

Concerning the association among the independent variables and both outcomes, oral motor functional performance and nutritional status, the Poisson regression analysis was performed. The significant variables (<0.20) were included in a multivariate regression model.

The Poisson’s regression analysis and Spearman’s correlation were performed for data evaluation by using Stata 9.0 (Stata Corp LP, College Station, USA). The level of significance was set as 5 % for all tests used.

## Results

### Population characteristic

Tables 1 and 2 show the mean values ± standard deviation or *n* (%), respectively, for the socioeconomic and clinical variables, for both control and study groups.

**Table 1:** Clinical variables for the control and study group (*n* = 199)

	Study group (*N* = 70)	Control group (*N* = 129)
Independent variables	*N* (%) or Mean ± SD	*N* (%) or Mean ± SD
OMAS	2.21 ± 0.71	3 ± 0
NOT-S	6.5 ± 0.71	1.71 ± 0.71
Gender		
male	37 (52.9)	69 (53.49)
female	33 (47.1)	60 (46.51)
Age	11.81 ± 3.09	11.5 ± 3
Birth^a, b^		
Fullterm	34 (49.28)	112 (86.82)
preterm	35 (50.72)	17 (13.18)
Birthweight^a, c^		
normal	32 (46.38)	114 (88.37)
low	16 (23.19)	12 (9.3)
very low weight	21 (30.43)	3 (2.33)
Type of CP		
spastic	51 (72.86)	0
dystonic and mixed	19 (27.14)	0
GMFCS		
I	1 (1.43)	129 (100)
II	26 (37.14)	0
III	1 (1.43)	0
IV	3 (4.29)	0
V	39 (55.71)	0
Nutritional status		
eutrophy	35 (50)	91 (70.54)
thinness	4 (5.71)	4 (3.1)
severe thinness	4 (5.71)	0
overweight	15 (21.43)	21 (16.28)
obesity	5 (7.15)	11 (8.53)
severe obesity	7 (10)	2 (1.55)
Use of medication (yes)	51 (72.86)	5 (3.88)
Medication		
anticonvulsant	31 (44.29)	0
tranquilizer	20 (28.57)	1 (0.78)
others	22 (31.43)	4 (3.1)
Feeding		
alone	35 (50)	129 (100)
needs help	27 (38.57)	0
gastric probe	8 (11.43)	0
Impaired health (yes)		
sight	36 (51.43)	24 (18.6)
listening	8 (11.43)	2 (1.55)
respiratory system	21 (30)	38 (29.46)
cardiovascular system	0	1 (0.78)
others	1 (1.43)	2 (1.55)
Cognitive		
normal	0	129 (100)
mild-moderate disorder	37 (52.86)	0
severe disorder	33 (47.14)	0

*SD*: mean standard deviation

^a^1 responsible for the study group did not inform about birth weight

^b^Fullterm: between 37 and 41 weeks pregnant, preterm: < 37 weeks

^c^Normal: 2.5 Kg to 4Kg, low: < 2.5Kg to 1.5Kg, very low weight: <1.5 Kg

**Table 2:** Socioeconomic variables for the control and study group

	Study group (*N* = 70)	Control group (*N* = 129)
Independent variables	*n* (%) or Mean ± SD	*n* (%) or Mean ± SD
Caretaker with health impairment^a^	20 (28.57)	26 (20.15)
Caregiver - mother	58 (82.86)	122 (94.57)
Caregiver’s age	38.05 ± 6.36	38.78 ± 10.6
Father’s age	42.88 ± 6.36	41.2 ± 8.48
Aglomeration	1.08 ± 0.06	1.01 ± 0
Home		
ownership	53 (75.71)	102 (79.07)
rented and donated	17 (24.29)	27 (20.93)
Mother’s marital status		
partnership	40 (57.14)	102 (79.07)
no partnership	30 (42.86)	27 (20.93)
Number of children	2.18 ± 1.41	2.58 ± 2.83
Parents school degree^b^ (mother/father)		
≤ 8 years	45 (64.3)**/**45 (67.16)	75 (60)**/**65 (56.03)
> 8 years	25 (35.71)**/**22 (32.83)	50 (40)**/**51 (43.97)
Employed^c^		
mother	23 (33.33)	66 (52.8)
father	55 (87.3)	100 (87.72)
Full time occupation		
mother	15 (21.43)	48 (37.2)
father	45 (71.43)	96 (74.42)
Earned income (brazilian minimum wages)	2.7 ± 1.25	2.9 ± 0.57

*SD*: mean standard deviation

^a^Caregiver mentioned having health problem

^b^In the study group, 3 guardians did not know father’s school degree, in the control group 4 guardians did not know mother’s school degree and 13 father’s school degree

^c^In the study group, 5 could not inform mother’s occupation and 7 father’s occupation. In the control group 4 could not inform mother’s occupation and 15 father’s occupation

### Oral motor evaluation

The correlation values between oral motor evaluation indexes showed statistically significant (*P* < 0.0001) but inversely proportional (*r* = -0.439; 95 % CI = -0.545 to -0.320) results.

Univariate Poisson’s regression analysis demonstrated association between the oral motor evaluation methods (OMAS and NOT-S) for the groups, between the forms of CP and for the GMFCS (≥4). There was association between dystonic and mixed types only for the NOT-S method.

With regards to the groups, 26 % of the evaluated population of CP patients demonstrated worst scores for the OMAS, and 3 times greater for the NOT-S, compared to the healthy patients. Within the CP patients, the spastic group demonstrated the smallest scores for the OMAS and a greater level of impairment for the NOT-S, as well as for the dystonic and mixed forms (Table 3).

**Table 3:** Univariate Regression Analysis for the association between the independent variables and the oral motor evaluation methods

	OMAS		NOT-S	
Variables	RR (95 % CI)	P*	RR (95 % CI)	P*
Group Control group	0.74 (0.65–0.84)	<0.001	3.79 (3.33–4.31)	<0.001
CP form				
spastic	0.67 (0.56–0.79)	<0.001	3.90 (3.40–4.47)	<0.001
Dystonic and mixed	0.93 (0.83–1.04)	0.261	3.50 (2.89–4.23)	<0.001
GMFCS	0.87 (0.82–0.91)	<0.001	1.34 (1.29–1.39)	<0.001

RR = *rate ratio*; 95 % CI = 95 % confidence interval

*Calculated by the Wald’s test

### Nutritional status evaluation

In our study, Poisson Univariate regression analysis showed significant results for some of the independent variables, considering that children were above the weight (nutritional evaluation). A multivariate model was designed considering the significant variables previously observed (Table 4).

**Table 4:** Possion’s Final Regression Model used to evaluate the associations between the independent variables for the overweight outcome

Variables	PR ajusted (95 % CI)	P*
Mother’s marital status (with partnership)		
Without partnership	0.57 (0.33–0.98)	0.045
Respiratory system (Normal)		
Impaired	1.63 (0.97–2.74)	0.062
Cardiovascular system (Normal)		
Impaired	5.36 (0.71–40.03)	0.101
OMAS (Continuous Variable)	2.46 (1.10–5.53)	0.028
CP form (control group)		
spastic	1.72 (0.90–3.29)	0.100
Dystonic and mixed	2.12 (1.05–4.26)	0.034

*Calculated by the Wald’s test

PR = prevalence ratio; 95 % CI = 95 % ConfidenceInterval

Variables in parenthesis refers to the reference group

Table 4 shows the association for overweight variables and demonstrates that patients with CP, whose mother reported having no partnership, showed a greater prevalence of being overweight, as well as the ones who presented mild oral motor impairment, given by the evaluation scores (OMAS). Regarding the CP forms, dystonic and mixed were statistically significant, pointing that there is a greater prevalence for those who are overweight.

In the Poisson’s Univariate regression analysis of the nutritional status evaluation for underweight children/adolescents, the variables which demonstrated significance were those related with oral motor dysfunction (OMAS and NOT-S), birth weight, use of medication (anticonvulsant, tranquilizer), feeding, cognitive impairment, GMFCS and father’s occupation (Table 5). Though, when designing the multivariate model, the variables lose significance.

**Table 5:** Poisson’s Univariate Regression Analysis for the association between the independent variables and the underweight condition

Variables	Crude PR (95 % CI)	P*
OMAS (continuous variable)	0.44 (0.31–0.62)	<0.001
NOT-S (continuous variable)	1.19 (1.03–1.36)	0.014
Birth (term)		
preterm	2.80 (0.94–8.34)	0.063
Birth weight (normal)		
Low weight + very low weight	3.93 (1.30–11.87)	0.015
Very low weight (low weight)	1.79 (1.05–3.05)	0.031
Anticonvulsant	3.87 (1.30–11.45)	0.014
Tranquilizer	4.23 (1.39–12.91)	0.011
Feeding (eating by him/herself) with help + sonda	4.03 (2.27–7.17)	<0.001
Feeding help (normal)	3.64 (0.87–15.24)	0.077
Probe (normal)	16.4 (4.40–61.07)	<0.001
Cognitive impairment (moderate) Severe	2.81 (1.47–5.38)	0.002
GMFCS (continuous variable)	1.68 (1.22–2.31)	0.001
Mother’s School degree (≤ 8 years)		
>8 years	0.14 (0.01–1.10)	0.063
Father’s occupation (no)		
yes	14.15 (8.64–23.17)	<0.001

*Calculated by the Wald’s test

PR = prevalence ratio; 95 % CI = 95 % ConfidenceInterval

## Discussion

The oral motor evaluation was performed by means of the OMAS instrument and demonstrated that patients with CP have a greater prevalence of lower scores (less ability) when compared tohealthy patients. Meanwhile, the NOT-S instrument, showed higher scores, which reveals greater impairment.

Among the type of CP patients, the spastic ones were the group presenting greater oral motor impairment. This might have occurred because spastic CP children/adolescents were the ones with a greater level of impairment and demonstrated difficulties to cooperate or did not understand the reproducibility of the movements, which reinforces the advantage of using the OMAS instrument in these conditions. An analysis of both scales, based on our clinical experience, showed that the OMAS can be more recommended as it does not depend upon patient’s cooperation, understanding of required movements and direct interaction between patient-examiner throughout the evaluation. Additionally, the mean time spent to complete the OMAS analysis was smaller compared to the other scale.

For the gross motor function the greater the score on the scale (≥4), the greater the orofacial motor impairment, demonstrating that CP may lead to a greater impairment of oral motor facial function.

Beyond the correlation between the methodologies, we tried to associate orofacial motor function with the nutritional status of children/adolescents with CP.

Studies in nutritional area evaluating problems related to nutrition in people with neuromotor disability have been mainly performed in developed countries [22–24], and have been scarce in developing countries.

Among the literature findings, there are also some studies reporting the results of underweight children [14]. Though, Rogozinski et al. [5] reported that the obesity prevalence within ambulatory CP children increased in the USA, from 7.7 % during the period of 1994–1997 to 16.5 % in between 2003 and 2004. Know et al. [25] also reported the prevalence of obesity and being overweight to be 14.6 % for the ambulatory patients with CP in Korea, but they did not show an increase along the recent years. Moreover, it has been reported in the literature that weight excess and the risk of weight excess between CP patients are greater than in general population [26].

Therefore, our results are in agreement with those findings, unraveling that out of the eutrophic condition, the majority of the CP population was overweight. The possible causes could be little or non-existent physical activity between CP children/adolescents, due to physical limitations, lack of accessibility at school playground and/or in the gym, cognitive difficulties which can also affect the interaction within those activities, or even tiring and muscle pain, leading to sedentary lifestyle [22]. Among those patients, dystonic and mixed forms demonstrated a greater prevalence to be overweight, probably due to the mild orofacial motor impairment feature and also because they can feed themselves with less trouble.

Family structure, or parental union status, is an important aspect of family context which has been associated to the development of many children. However, very little is known about how the family structure can affect nutritional health of children [27, 28]. It seems that children with stable single mothers have larger gains in BMI and higher risk of becoming overweight or obese [28]. According to Schmeer [28], the study models for BMI show that economic resource changes due to family dissolution, less social or emotional support and a smaller participation of parents can lead to an increase in child’s stress during this transition period, thus, resulting in an increase in BMI, and consequently a greater risk of becoming overweight/obese.

With regards to thinness or severe thinness, having better oral motor ability (greater scores for OMAS and smaller scores for NOT-S) and better gross function were identified as a protective factor, as the feeding improves, and less problems caused by poor nutrients intake occur. The association with severe impaired cognition may occur because of the difficulties faced by caregivers in knowing the right demand of food, time between meals and the amount required. Despite the small number of children fed by means of a probe, there was a strong association between the fact of being underweight and this way of nutrition. Calis et al. [29] also observed an association of tube feeding and lower anthropometric Z-scores.

Although the research has reached its aims, there were some limitations that occurred due to individual’s intellectual impairment to cooperate during evaluation. Besides, this is an observational study with a convenience sample, which may have influenced the results. More studies should be done with higher number of underweight patients.

## Conclusions

From the data collected, we could conclude that:

- There is significant correlation between oral motor evaluation instruments OMAS and NOT-S;
- A better oral facial functional performance favors the gain of weight in CP children and adolescents, independent on the gross motor function. Dystonic CP children/adolescents presented mild impairment of the oral motor function, compared to the spastic ones.
- There is a significant association between nutritional status and mother’s marital status, which suggest that social factors may interfere with the assessed outcome.
